# Factors associated with actionable gene aberrations in pancreatic cancer based on the C-CAT database

**DOI:** 10.1007/s00535-025-02253-9

**Published:** 2025-05-02

**Authors:** Go Endo, Kazunaga Ishigaki, Yousuke Nakai, Hiroto Nishio, Koshiro Fukuda, Kota Ishida, Shinya Takaoka, Yurie Tokito, Rintaro Fukuda, Kensaku Noguchi, Hiroki Oyama, Tatsunori Suzuki, Tatsuya Sato, Tomotaka Saito, Tsuyoshi Hamada, Koji Miyabayashi, Naminatsu Takahara, Yasuyoshi Sato, Hidenori Kage, Katsutoshi Oda, Mitsuhiro Fujishiro

**Affiliations:** 1 Department of Gastroenterology, Graduate School of Medicine, The University of Tokyo, 7-3-1 Hongo Bunkyo-ku, Tokyo, 113-8655 Japan; 2 Department of Clinical Oncology, The University of Tokyo Hospital, Tokyo, Japan; 3 Department of Endoscopy and Endoscopic Surgery, The University of Tokyo Hospital, Tokyo, Japan; 4 Department of Hepato-Biliary-Pancreatic Medicine, The Cancer Institute Hospital, Japanese Foundation for Cancer Research, Tokyo, Japan; 5 Department of Respiratory Medicine, The University of Tokyo, Tokyo, Japan; 6 Department of Clinical Genomics, The University of Tokyo, Tokyo, Japan

**Keywords:** Pancreatic cancer, Comprehensive genomic profiling test, *KRAS*

## Abstract

**Background:**

Comprehensive genomic profiling (CGP) tests are increasingly used to explore the genomically matched therapies for solid tumors. The aim of this study is to investigate factors associated with actionable gene aberrations in pancreatic cancer (PC) using real-world data from the Center for Advanced Cancer Genome Therapy (C-CAT) database.

**Methods:**

Among 6768 patients with unresectable and recurrent PC registered in the C-CAT database between June 2019 and July 2023, we identified 4628 patients who underwent tissue-based CGP tests using either FoundationOne^®^ CDx (F1CDx) or OncoGuide^™^ NCC Oncopanel (NOP). We investigated the incidence of actionable gene aberrations and the factors associated with their detection.

**Results:**

The cohort included 3,554 patients who underwent F1CDx and 1128 NOP, with surgical specimens in 50% of the cases. Adenocarcinoma was the predominant subtype (95%), and *KRAS* mutations were found in 90%. The overall incidence of actionable gene aberrations was 27%. The most common gene abnormalities were *BRCA2* (3.4%), followed by *ATM* (2.9%), *ERBB2* (2.8%), *PIK3 CA* (2.5%), and *BRAF* (1.9%). Multivariable analysis revealed that acinar cell carcinoma (ACC) (Odds ratio [OR] 1.87, 95% confidence interval [CI] 1.00–2.67), *KRAS* wild type (*KRAS*_WT_) (OR 3.09, 95% CI 2.49–3.85), and use of F1CDx (OR 2.38, 95% CI 1.98–2.85) were significantly associated with actionable gene aberrations.

**Conclusions:**

Actionable gene aberrations were more likely in cases of ACC, *KRAS*_WT_, and F1CDx usage. The choice of CGP test should be made on a case-by-case basis, as other factors beyond actionable gene aberrations also need to be considered.

## Introduction

The incidence of pancreatic cancer (PC) is on the rise, and it is expected to become the second leading cause of cancer-related deaths in the United States by 2040 [1, 2]. While PC is one of the most lethal malignancies, its prognosis has slowly but steadily improved largely due to advancements in palliative chemotherapy which is recommended for the vast majority of patients. Currently, combination chemotherapy with cytotoxic agents, such as FOLFIRINOX (FFX) [3] and gemcitabine plus nab-paclitaxel (GnP) [4], is a mainstay for advanced disease, but there are concerns about potential harms. Therefore, it is desirable to develop novel, tailored treatments based on the specific genetic profile of each tumor to further improve patient outcomes.

Recently, comprehensive genomic profiling (CGP) tests have emerged as a pivotal tool in the clinical management of cancer, offering the potential to inform personalized treatments [5–7]. In PC, matched therapy based on actionable gene abnormalities has been shown to improve prognosis [8]. In addition to guiding therapy, CGP tests can provide valuable insights into carcinogenesis and contribute to a better understanding of the molecular mechanisms underlying the disease, which vary between individuals. In Japan, two tissue-based CGP tests, FoundationOne^®^ CDx (F1 CDx) [6] and OncoGuide^™^ NCC Oncopanel (NOP) [5], were first introduced into clinical practice in 2019 and are now increasingly used for patients with solid tumors who have failed or are expected to be refractory to standard treatment. The results of all CGP tests are registered in a nationwide database called the Center for Advanced Cancer Genome Therapy (C-CAT) to support innovative research and the development of new treatments [9, 10]. Research using the C-CAT database has already been reported for PC, including studies of genetic abnormalities by histological subtype and the efficacy of chemotherapy [11, 12].

A significant challenge of CGP tests is the limited accessibility to matched therapies (< 5%) [13]. Given the high medical costs associated with CGP tests, it is crucial to identify patients who are more likely to harbor actionable gene aberrations directly linked to treatment. Previous studies have indicated that patients with acinar cell carcinoma (ACC) [14, 15] or *KRAS* wild type (*KRAS*_WT_) [16, 17] have a higher probability of harboring actionable gene aberrations. However, these studies did not specifically focus on genetic aberrations with treatment targets validated by clinical studies. Consequently, it remains uncertain whether these findings can effectively contribute to improving access to targeted therapies in real-world clinical practice. To address this gap, this study aimed to investigate incidence and factors associated with actionable gene aberrations supported by evidence levels provided in the C-CAT database.

## Methods

### Study population

**Fig. 1:**
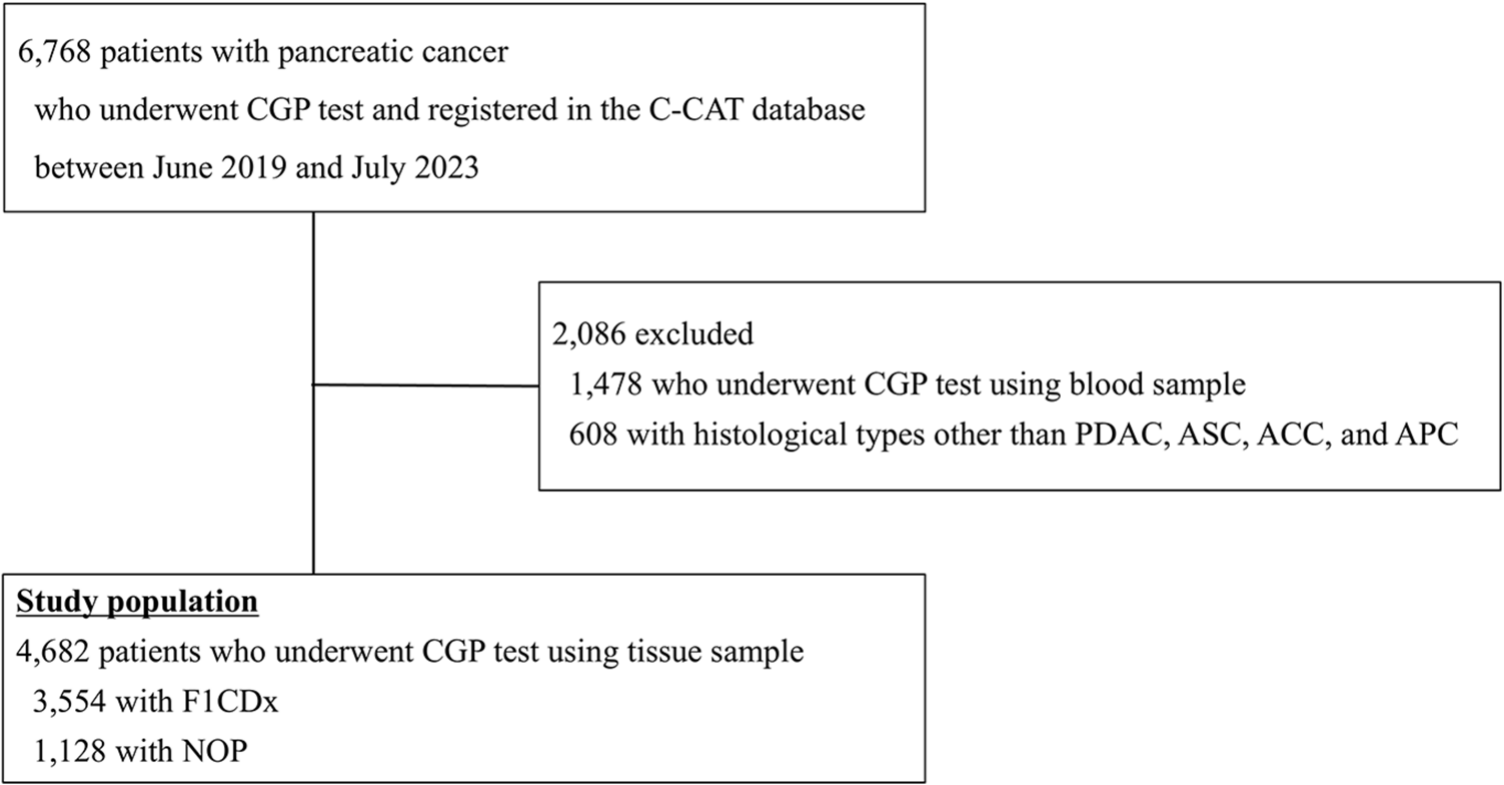
The study population comprised 6,768 cases from the Center for Cancer Genomics and Advanced Therapeutics (C-CAT) database collected at the end of July 2023. In total, 4,682 cases were included, and the details of their histology are shown in the diagram. *ACC* acinar cell carcinoma, *APC* anaplastic carcinoma, *ASC* adenosquamous carcinoma, *PDAC* pancreatic ductal adenocarcinoma

This is a retrospective observational study using data from the C-CAT database, which includes genomic information from CGP tests and manually entered clinical information. Between June 2019 and July 2023, 6768 patients with unresectable and recurrent PC were registered in the C-CAT database. Among them, 1478 patients who underwent liquid-based CGP tests were excluded due to the limited gene mutation detection rate compared to that of tissue-based CGP tests. In addition, we excluded 608 patients with histology other than pancreatic ductal adenocarcinoma (PDAC), adenosquamous carcinoma (ASC), ACC, and anaplastic carcinoma (APC). Ultimately, 4,682 patients who underwent F1 CDx or NOP were analyzed in this study (as shown in Fig. 1).

This study was conducted according to the guidelines in the Helsinki Declaration and was approved by the ethics committee of The University of Tokyo (# 2021341G) and the Information Utilization Review Board of C-CAT (# CDU2022-026 N).

### Sequencing and definition of actionable gene aberration

F1 CDx assays single-base substitutions, insertions, deletions, copy number alterations, and rearrangements in 324 genes. F1 CDx reports these gene abnormalities at variant allele frequencies (VAF) ≥ 5% and at VAF ≥ 1% in hotspot regions [6]. The NOP assay examines these gene abnormalities in 124 genes at VAF ≥ 5% [5]. Both tests measure genomic signatures such as microsatellite instability (MSI) and tumor mutational burden (TMB). As F1 CDx only samples tumor tissue, it is not possible to distinguish between somatic and germline mutations. In contrast, NOP can distinguish between somatic and germline mutations by analyzing both tissue and blood samples. For F1 CDx, a tissue sample ≥ 25 mm^2^ and a tumor content rate ≥ 30% are recommended for appropriate analysis, and ≥ 16 mm^2^ and ≥ 20% for NOP.

Evaluation of pathogenicity was added to each gene aberration according to the clinical practice guidelines for next-generation sequencing in cancer diagnosis and treatment [18]. The guidelines provide the following levels of evidence for each gene aberration: level A, a Pharmaceuticals and Medical Devices Agency- or Food and Drug Administration-approved biomarker for the tumor type or a biomarker verified by a prospective molecularly driven clinical trial; level B, a biomarker identified via subgroup analysis in a prospective clinical trial; level C, an approved biomarker for a different tumor type or a biomarker with evidence supporting its clinical utility; level D, a biomarker with evidence of proof of concept in at least one case report; level E, a biomarker with evidence obtained from in vitro/in vivo experiments.

In this study, gene abnormalities with evidence levels A–D were considered actionable. TMB-H was defined as 10 Mut/Mb or more [19]. Actionable gene aberrations included cases of MSI-H and TMB-H, in addition to actionable gene abnormalities. For *KRAS*, only the G12 C-mutant was defined as actionable because *KRAS* G12 C inhibitor is currently available for tumors with this mutation [20, 21].

### Data collection

Clinical information, including sex, age, Eastern Cooperative Oncology Group performance status (ECOG PS), drinking and smoking history, presence of double cancer, family history of cancer, histology, metastatic organs, and treatment before sampling, was collected. Information on tissue sampling, including sampling site (primary or metastatic tumor) and method (surgery or biopsy), was also collected. The background information for each CGP test was compared. Genomic information included genes detected as abnormal, the mutation type, VAF, evidence level, MSI, and TMB status. No distinction was made between germline and somatic mutations in this study. Due to the nature of the C-CAT database, which is based on data manually entered by attending physicians, some cases had missing data. Therefore, the rates for each variable were based on the number of patients for whom data were available. In the C-CAT database, unknown information is categorized as “unknown,’ and the frequency of missing values for the variables used in this analysis was relatively low, ranging from 0.1 to 4.7%.

### Statistical analysis

Continuous variables are presented as median with range and compared using the Mann**–**Whitney *U* test. Categorical variables are presented as numbers with percentages and compared using Fisher’s exact test. Factors associated with the actionable gene aberrations were examined via univariate and multivariable logistic regression in each CGP test. Factors with *P* ≤ 0.10 in the univariate analyses were included in the multivariable logistic regression analyses.

In order to adjust the differences in sample volume and VAF threshold between the two CGP test, additional analyses were conducted to explore factors associated with actionable gene aberrations in surgical and biopsy samples, considering only mutations with VAF ≥ 5% as relevant.

All statistical tests were two-tailed and the significance level was set at a *P* value of 0.05. Stata version 18.0 (StataCorp, College Station, TX, USA) was used for all statistical analyses.

## Results

### Patient characteristics

Patient characteristics are summarized in Table 1. In 4682 cases, 3554 underwent F1 CDx (76%) and 1128 underwent NOP (24%). Adenocarcinoma was the predominant histological subtype accounting for 95%. There were no significant differences in patient characteristics between the two CGP test groups, except for sex distribution (56% male in F1 CDx vs. 60% in NOP, *P* < 0.001). In addition, the proportions of surgical specimens (55% in F1 CDx vs. 36% in NOP, *P* < 0.001) and previously treated specimens (43% in F1 CDx vs. 36% in NOP, *P* < 0.001) were different between the two groups.

**Table 1:** Clinical characteristics

		Total	F1 CDx	NOP	*P* value
		*n* = 4682	*n* = 3554	*n* = 1128	
Sex	Male	2674 (57)	1992 (56)	682 (60)	**0.016**
Age	Median (range), years	66 (5–88)	66 (5–88)	66 (24–86)	0.31
ECOG PS	0/1/2–4	2775 (61)/1647 (37)/82 (2)	2151 (63)/1205 (35)/68 (2)	624 (58)/442 (41)/14 (1)	0.059
Smoking history	Yes	2211 (49)	1675 (49)	536 (49)	0.83
Drinking history	Yes	595 (14)	459 (14)	136 (13)	0.29
Double cancer (different organs)	Yes	468 (10)	353 (10)	115 (11)	0.080
Family history of cancer	Yes	3330 (73)	2537 (74)	793 (72)	0.72
Histology	PDAC	4465 (95)	3383 (95)	1082 (96)	0.78
	ASC	106 (2)	85 (2)	21 (2)	
	ACC	80 (2)	62 (2)	18 (2)	
	APC	31 (1)	24 (1)	7 (1)	
Metastasis	Yes	4087 (91)	3106 (91)	981 (90)	0.63
Sampling area	Primary	3415 (73)	2583 (73)	832 (74)	0.51
	Metastasis	1265 (27)	969 (27)	296 (26)	
Sampling method	Surgery	2326 (50)	1923 (55)	403 (36)	** < 0.001**
	Biopsy	2317 (50)	1602 (45)	715 (64)	
Treatment before sampling	Yes	1534 (41)	1244 (43)	290 (36)	** < 0.001**

Bold values indicate statistically significant differences (P 0.05)

Data are shown in number (%) or median (range)

The total number of cases varies due to missing data in certain variables

*ACC* acinar cell carcinoma, *APC* anaplastic carcinoma, *ASC* adenosquamous carcinoma, *ECOG PS* Eastern Cooperative Oncology Group Performance Status, *F1 CDx* FoundationOne CDx^®^, *NOP* OncoGuide^™^ NCC Oncopanel, *PDAC* pancreatic ductal adenocarcinoma

### Details of actionable gene aberrations

The breakdown of actionable gene aberrations and the 10 most common actionable abnormalities are shown in Table 2. The 10 most common genetic abnormalities are shown in Supplementary Table 1. In total, actionable gene aberrations were observed in 1,264 cases (27%). The most common actionable gene abnormalities were *BRCA2* (3.4%), followed by *ATM* (2.9%), *ERBB2* (2.8%), *PIK3 CA* (2.5%), and *BRAF* (1.9%), in addition to MSI-H (0.5%) and TMB-H (2.4%). The median (range) TMB was 2.3 (0–281.4) mut/Mb. Actionable gene aberrations were more likely to be observed in F1 CDx than in NOP (30% vs. 18%, *P* < 0.001). The frequency of MSI-H was not different between the two tests (0.5% vs. 0.4%, *P* = 0.80), but the TMB-H detection rate was higher in NOP (1.7% vs. 4.3%, *P* < 0.001). The most common gene abnormality, regardless of evidence level, was *KRAS* (88.4%) (Supplementary Table 1).

**Table 2:** The breakdown of actionable gene aberrations and the 10 most common actionable abnormalities

	Total	F1 CDx	NOP	*P* value
	*n* = 4682	*n* = 3554	*n* = 1128	
Actionable gene aberration	1264 (27.0)	1063 (29.9)	201 (17.8)	** < 0.001**
MSI-H	20 (0.4)	16 (0.5)	4 (0.4)	0.80
TMB-H	111 (2.4)	62 (1.7)	49 (4.3)	** < 0.001**
Actionable abnormality				
*BRCA2*	158 (3.4)	119 (3.4)	39 (3.5)	0.59
*ATM*	134 (2.9)	111 (3.1)	23 (2.0)	0.088
*ERBB2*	131 (2.8)	99 (2.8)	32 (2.8)	0.99
*PIK3 CA*	118 (2.5)	90 (2.5)	28 (2.5)	0.93
*BRAF*	91 (1.9)	74 (2.1)	17 (1.5)	0.90
*FGFR1*	87 (1.9)	70 (2.0)	17 (1.5)	0.34
*PALB2*	63 (1.3)	47 (1.3)	16 (1.4)	0.76
*MDM2*	45 (1.0)	31 (0.9)	14 (1.2)	0.24
*PTEN*	44 (0.9)	34 (1.0)	10 (0.9)	0.46
*BRCA1*	43 (0.9)	36 (1.0)	7 (0.6)	0.15

Bold values indicate statistically significant differences (P 0.05)

Data are shown in number (%)

*MSI-H* microsatellite instability-high, *TMB-H* Tumor mutational burden-high

### Factors associated with the actionable gene aberrations

The results of univariate and multivariable logistic regression for factors associated with actionable gene aberrations detected by CGP tests are shown in Table 3. In the univariate analyses, age (< 40 years), double cancer (presence of another tumor), the histology of ACC, sampling from metastasis, sampling by biopsy, *KRAS*_WT_ status, and use of F1 CDx were associated with the detection of actionable gene aberrations. Multivariable analysis revealed the histology of ACC (Odds ratio [OR] 1.87, 95% confidence interval [CI] 1.00–2.67, *P* = 0.043), *KRAS*_WT_ status (OR 3.09, 95% CI 2.49–3.85, *P* < 0.001), and use of F1 CDx (OR 2.38, 95% CI 1.98–2.85, *P* < 0.001) as significant factors associated with the detection of actionable gene aberrations.

**Table 3:** Univariate and multivariable logistic regression of factors associated with actionable gene aberrations

	Univariate analysis			Multivariable analysis	
	*n*	Actionable gene aberration	*P* value	OR (95% CI)	*P* value
Sex					
Male	2674	715 (26.7)	0.64		
Female	2007	549 (27.4)			
Age					
< 40 years	64	26 (40.6)	**0.016**	1.52 (0.88–2.63)	0.14
≥ 40 years	4613	1236 (26.8)			
ECOG PS					
0–1	4422	1186 (26.8)	0.32		
2–4	82	26 (31.7)			
Smoking					
Yes	2211	584 (26.4)	0.38		
No	2190	605 (27.6)			
Alcohol					
Yes	595	158 (26.6)	0.73		
No	3549	971 (27.4)			
Double cancer					
Yes	468	145 (31.0)	**0.048**	1.12 (1.01–1.24)	0.061
No	4033	1072 (26.6)			
Family history					
Yes	3330	914 (27.4)	0.33		
No	1101	285 (25.9)			
Histology					
ACC	80	44 (55.0)	** < 0.001**	**1.87 (1.00**–**2.67)**	**0.043**
Others	4602	1220 (26.5)			
Metastasis					
Yes	4087	1107 (27.1)	0.99		
No	394	106 (26.9)			
Sampling area					
Primary	3415	863 (25.3)	** < 0.001**		
Metastatic	1265	400 (31.6)		1.18 (0.99–1.37)	0.057
Sampling method					
Surgery	2326	554 (23.8)	** < 0.001**		
Biopsy	2317	697 (30.1)		1.43 (0.94–1.34)	0.13
Treatment before sampling					
Yes	1534	430 (28.0)	0.91		
No	2172	605 (27.8)			
CGP test					
F1 CDx	3554	1063 (29.9)	** < 0.001**	**2.38 (1.98**–**2.85)**	** < 0.001**
NOP	1128	201 (16.8)			
*KRAS* mutation					
Yes	4218	1038 (24.6)	** < 0.001**		
No	464	226 (48.7)		**3.09 (2.49**–**3.85)**	** < 0.001**

Bold values indicate statistically significant differences (P 0.05)

Data are shown in number (%)

The total number of cases varies due to missing data in certain variables

*ACC* acinar cell carcinoma, *CGP* comprehensive genetic profiling, *CI* confidence interval, *ECOG PS* Eastern Cooperative Oncology Group Performance Status, *F1 CDx* Foundation One CDx^®^, *NOP* OncoGuide^™^ NCC Oncopanel, *OR* odds ratio

The results of the additional analysis for surgical and biopsy samples, considering mutations with a VAF ≥ 5% as relevant, are shown in Supplementary Tables 2 and 3, respectively. Consistent with the findings for the entire cohort, these additional analyses revealed that the histology of ACC, KRAS_WT_ status, and the use of F1 CDx were independent factors associated with actionable gene aberrations in the multivariate analyses.

### Actionable gene aberrations according to the histological type, *KRAS* status, and CGP tests

**Fig. 2:**
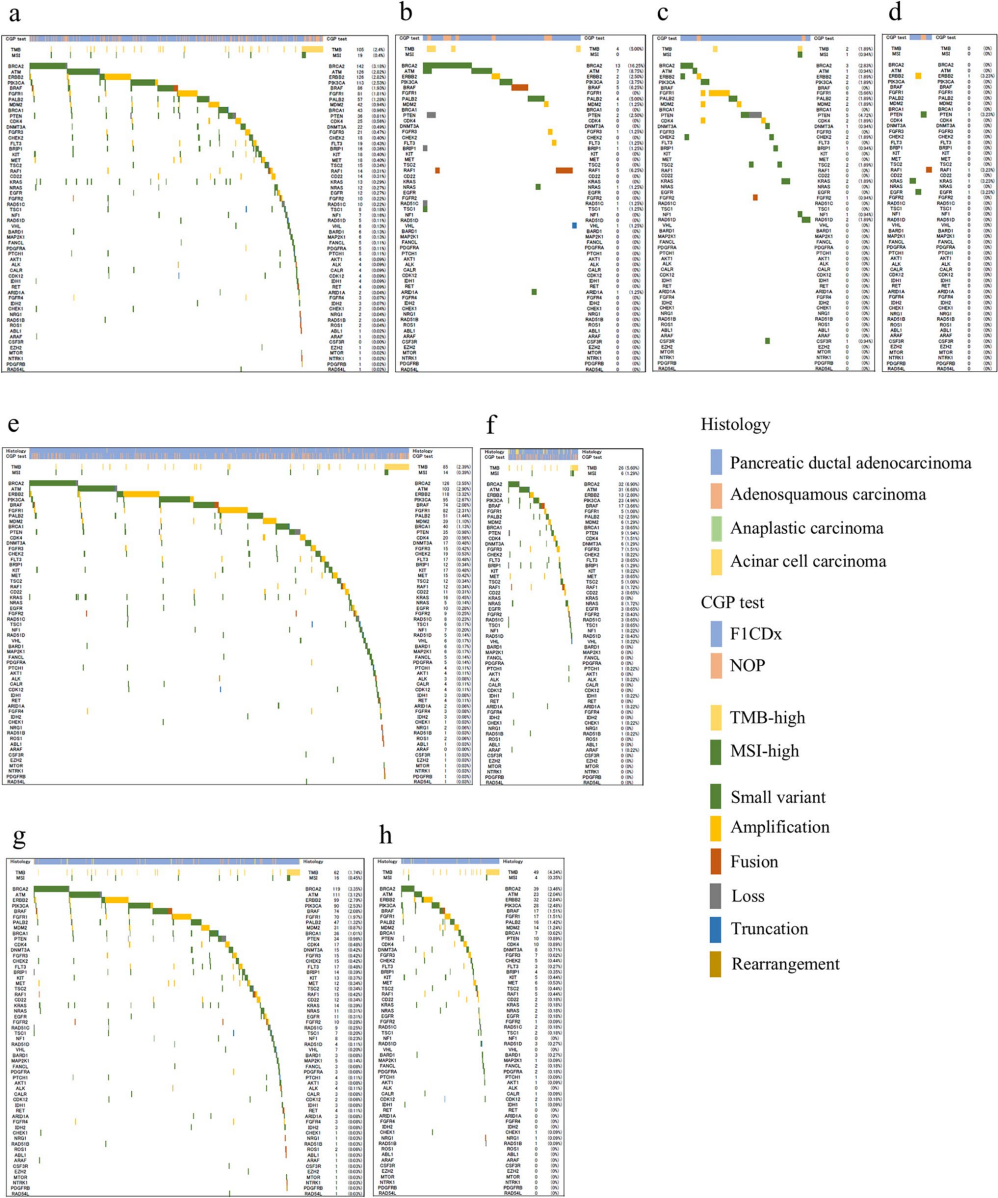
Summary of CGP tests submitted and actionable gene aberrations. **a** Pancreatic ductal adenocarcinoma. **b** Acinar cell carcinoma. **c** Adenosquamous carcinoma. **d** Anaplastic carcinoma. **e** *KRAS* mutant. **f** *KRAS* wild type. **g** FoundationOne CDx^®^. **h** OncoGuide^™^ NCC Oncopanel FoundationOne CDx

Details of actionable gene aberrations according to the histological type, *KRAS* status*,* and CGP test are shown in Fig. 2. Actionable gene aberrations were more common in ACC cases than in other histological types (55% vs 27%, *P* < 0.001). Furthermore, actionable *BRCA2* (16% vs 3%, *P* < 0.001), *ATM* (9% vs 3%, *P* < 0.001), *BRAF* (6% vs 2%, *P* < 0.001), and *PALB2* (5% vs 1%, *P* < 0.001) mutations were more frequent than in PDAC (Fig. 2b). A higher proportion of TMB-H (6% vs 2%, *P* < 0.001) and MSI-H (1.3% vs 0.3%, *P* = 0.010) mutations were observed in cases with *KRAS*_WT_ compared to *KRAS* mutant (*KRAS*_mt_). In addition, *KRAS*_WT_ cases were more likely to harbor mutations in *BRCA2* (7% vs 4%, P < 0.001), *PIK3 CA* (5% vs 3%, *P* < 0.001), *PALB2* (3% vs 1%, *P* = 0.032), *PTEN* (2% vs 1%, P = 0.042), and *BRIP1* (1.3% vs 0.3%, *P* = 0.002) compared to *KRAS*_mt_ cases (Fig. 2e, f). A higher incidence of fusion genes was noted in *KRAS*_*WT*_ cases, including *BRAF* (4% vs 0%, *P* < 0.001), *FGFR2* (1% vs 0%, *P* < 0.001), and *RAF1* (1% vs 0%, *P* < 0.001). The *KRAS* G12 C mutation was detected in 16 cases (0.4%).

There was no difference in the detection of actionable gene abnormalities between F1 CDx and NOP in a gene-by-gene comparison for all genetic abnormalities detected (Fig. 2g, h).

### Subgroup analysis between the two CGP tests

**Fig. 3:**
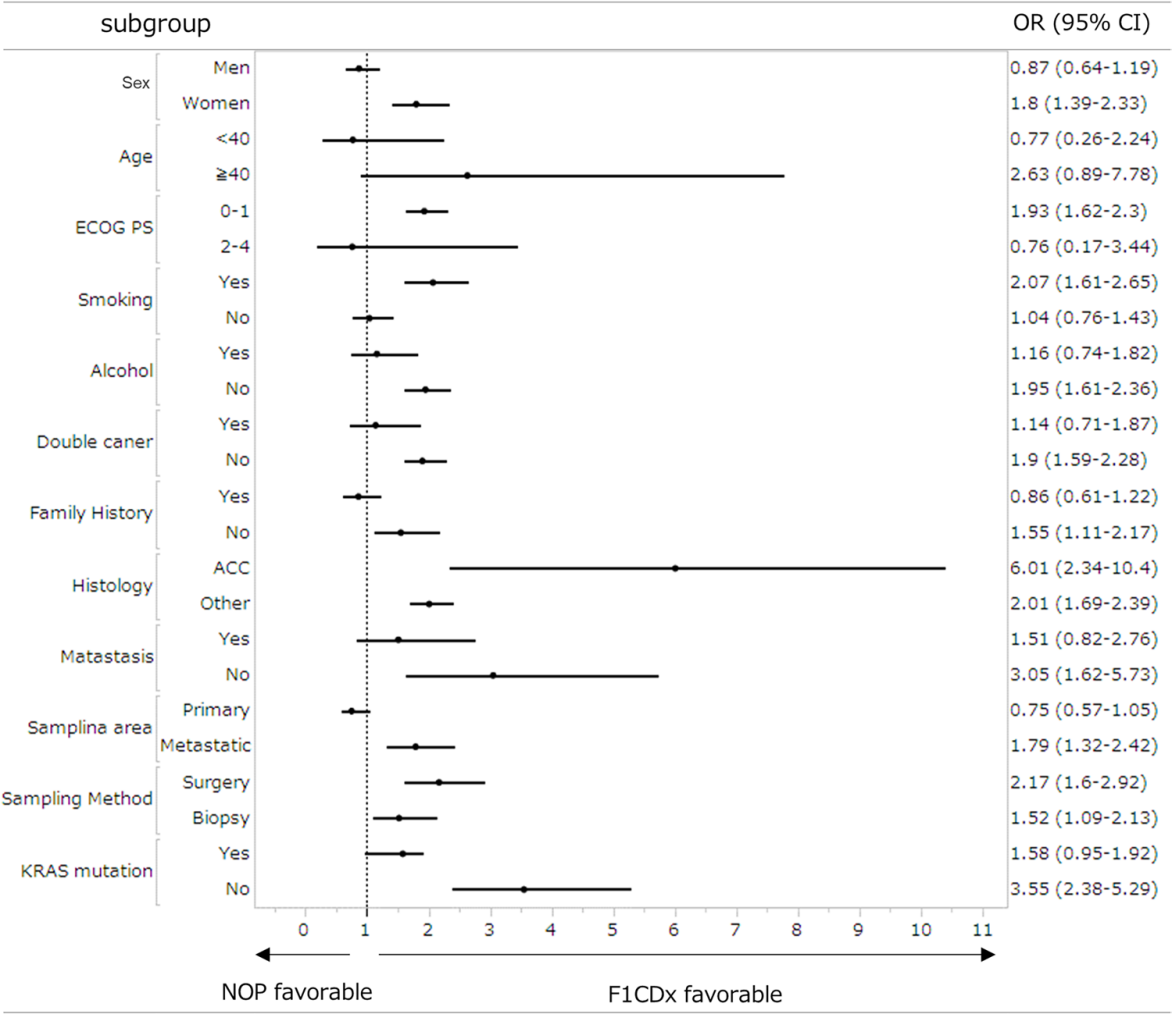
Forest plot for the detection rate of actionable gene aberration. Odds ratio in background factors for each test is shown

A forest plot illustrating the favorability of the two CGP tests in detecting actionable gene aberrations across subgroups is shown in Fig. 3. Despite differences in the recommended quantity and quality of submitted specimens for the two CGP tests, F1 CDx contributed to the detection of actionable gene aberrations in most groups, particularly in those with ACC (OR 6.01, 95% CI 2.34–10.4) and *KRAS*_WT_ (OR 3.55, 95% CI 2.38–5.29). No subgroups were identified in which NOP contributed to the detection of actionable gene aberrations.

## Discussion

In this study of the nationwide CGP test C-CAT database, we found that 1264 out of 4682 patients (27%) with unresectable and recurrent PC harbored actionable gene aberrations. There are some reports of the frequency of actionable gene aberrations in PC based on small numbers of cases, and there is no significant difference from our findings [22–24]. We demonstrated that the histology of ACC and *KRAS*_WT_ was significantly associated with actionable gene aberrations, which is consistent with trends reported in previous studies [11, 14–17]. In addition, our analysis showed that F1 CDx provided a higher probability of detecting actionable gene aberrations compared to NOP.

Our finding of a higher incidence of actionable gene abnormalities in ACC was consistent with previous studies. Florou et al. reported that ACC was significantly associated with a higher *BRCA2* mutation rate and a higher TMB than PDAC [14]. In addition, a previous analysis of C-CAT data showed that abnormalities in homologous recombination repair-related genes are common in ACC [11]. In this study, ACC demonstrated a higher frequency of gene aberrations such as *BRCA2* (16.3%) and *ATM* (8.8%), which may benefit from platinum-based therapy and *PARP* inhibitors [25, 26]. Although this study did not fully assess the accessibility to targeted therapies or their outcomes following CGP testing, the proactive use of CGP in ACC may enhance treatment outcomes through the delivery of tailored treatment.

Another key finding in this study is that *KRAS*_WT_ was significantly associated with actionable gene aberrations. *KRAS* is a key oncogene implicated in early tumorigenesis and detected in over 90% of PC cases [27, 28]. Several reports have identified differences in genetic abnormalities between *KRAS*_WT_ versus *KRAS*_mt_ cases [15–17]. This may be attributed to distinct genomic abnormalities promoting carcinogenesis in the absence of *KRAS* mutations. The results of the present study suggest that such abnormalities are often potential therapeutic targets. Further, KRAS inhibitors targeting mutations other than G12 C demonstrate promising activity and are currently under intensive investigation in both preclinical and clinical studies [20, 21]. Since the vast majority of patients with PC have KRAS mutation, and they may become candidates for KRAS inhibitors in the future.

Sample origin (primary vs. metastatic tumor) was not statistically significant in multivariable analysis but appeared marginally relevant in contributing to actionable gene aberrations (as shown in Table 3). Thus, the biological background and clinical implications of this finding should be discussed here. Previous studies have suggested that new genetic mutations may accumulate in metastatic lesions as the disease progresses [29–31]. Accordingly, genetic profiling using samples from metastatic tumors, rather than primary tumors, may more accurately reflect the tumor’s current status, including its heterogeneity and resistance to treatment, and may support the exploration of tailored therapeutic strategies. However, given the differences in treatment responsiveness between primary and metastatic lesions [32, 33], it remains unclear which sampling site for CGP tests offers higher accessibility to targeted therapies with improved clinical outcomes. Addressing these uncertainties represents a critical avenue for future research.

In this study, we demonstrated that F1 CDx was more likely to detect actionable gene aberrations as compared to NOP. This was true not only for the entire cohort but also in the additional analyses of surgical and biopsy samples, where only mutations with a VAF ≥ 5% were considered relevant (as shown in Table 3 and Supplementary Tables 2 and 3). One possible reason is the difference in the number of genes assessed and the VAF detection thresholds between the two tests. Another reason can be the difference in the appropriate specimen standards of sample size and VAF threshold. Due to differences in the criteria for submitted tissue samples, sampling method was different between the two CGP tests (Table 1). However, considering the lack of a clear distinction in treatment accessibility between the two tests [34], it is difficult to conclude that F1 CDx is superior to NOP based on our results. NOP has certain advantages over F1 CDx, including its suitability for relatively small tissue samples and its ability to identify germline mutations. In pancreatic cancer, BRCA gene mutations have been reported in 5.2% of cases, with approximately two-thirds being germline mutations and one-third somatic mutations [35]. The presence of BRCA mutations not only offers a treatment opportunity with PARP inhibitors in PC individuals but also necessitates surveillance for early detection of breast, ovarian, and prostate cancers, as well as follow-up care for at-risk family members. Thus, determining whether a BRCA mutation is germline or somatic is clinically significant. Although it was not evaluated in this study, the ability to identify germline mutations is a major strength of NOP. The choice of test should be made on a case-by-case basis, taking into account the characteristics of each CGP test as described above.

This study has several limitations. First, we did not analyze the number of cases who received matched therapy according to their gene aberrations, and overall survival due to missing or inaccurate data. Second, selection bias could not be excluded. This cohort included only patients who were expected to continue chemotherapy in the future. These are unavoidable due to the nature of the C-CAT database, warranting more detailed studies of different cohorts. Third, the reasons for selection between two CGP tests are unclear. It might include a certain number of cases that could not be submitted to F1 CDx due to small sample volume and were submitted to NOP. In addition, the unique advantage of NOP, the ability to differentiate between germline and somatic mutations, was not evaluated, and the effectiveness of NOP may have been underestimated.

In conclusion, the CGP test contributed to the detection of actionable gene aberrations in up to 27% of PC patients. This analysis showed that actionable gene aberrations were more likely to be found in cases of ACC and *KRAS*_WT_ using large-scale real-world data. In addition, patients who underwent F1 CDx had a higher likelihood of having an actionable gene aberration detected, but the choice of test should be made on a case-by-case basis. Further studies are needed to improve the accessibility to target therapies which could enhance survival.

## Abbreviations

ACC: Acinar cell carcinoma
APC: Anaplastic carcinoma
ASC: Adenosquamous carcinoma
C-CAT: Center for Advanced Cancer Genome Therapy
CGP: Comprehensive genomic profiling
dMMR: De novo mismatch repair deficiency
ECOG PS: Eastern Cooperative Oncology Group Performance Status
F1 CDx: Foundation One^®^ CDx
F1 Liquid: Foundation One^®^ CDx Liquid
FFX: FOLFIRINOX
GnP: Gemcitabine combined with nab-paclitaxel
HRD: Homologous recombination deficient
*KRAS*_mt_: *KRA**S* Mutant
*KRAS*_WT_: *KRA**S* Wild type
MSI-H: Microsatellite instability-high
NOP: OncoGuide™ NCC Oncopanel
OS: Overall survival
PC: Pancreatic cancer
PDAC: Pancreatic ductal adenocarcinoma
TMB-H: Tumor mutational burden-high
VAF: Variant allele frequencies

## References

[1] Huang L, Jansen L, Balavarca Y, et al. Stratified survival of resected and overall pancreatic cancer patients in Europe and the USA in the early twenty-first century: a large, international population-based study. BMC Med. 2018;16:125. 10.1186/s12916-018-1120-9. 30126408 10.1186/s12916-018-1120-9 PMC6102804

[2] Siegel RL, Miller KD, Wagle NS, et al. Cancer statistics, 2023. CA Cancer J Clin. 2023;73:17–48. 10.3322/caac.21763. 36633525 10.3322/caac.21763

[3] Conroy T, Desseigne F, Ychou M, et al. FOLFIRINOX versus gemcitabine for metastatic pancreatic cancer. N Engl J Med. 2011;364:1817–25. 10.1056/NEJMoa1011923. 21561347 10.1056/NEJMoa1011923

[4] Von Hoff DD, Ervin T, Arena FP, et al. Increased survival in pancreatic cancer with nab-paclitaxel plus gemcitabine. N Engl J Med. 2013;369:1691–703. 10.1056/NEJMoa1304369. 24131140 10.1056/NEJMoa1304369 PMC4631139

[5] Sunami K, Ichikawa H, Kubo T, et al. Feasibility and utility of a panel testing for 114 cancer-associated genes in a clinical setting: a hospital-based study. Cancer Sci. 2019;110:1480–90. 10.1111/cas.13969. 30742731 10.1111/cas.13969 PMC6447843

[6] Takeda M, Takahama T, Sakai K, et al. Clinical application of the FoundationOne CDx assay to therapeutic decision-making for patients with advanced solid tumors. Oncologist. 2021;26:e588–96. 10.1002/onco.13639. 33325566 10.1002/onco.13639 PMC8018334

[7] Kage H, Shinozaki-Ushiku A, Ishigaki K, et al. Clinical utility of Todai OncoPanel in the setting of approved comprehensive cancer genomic profiling tests in Japan. Cancer Sci. 2023;114:1710–7. 10.1111/cas.15717. 36601953 10.1111/cas.15717 PMC10067384

[8] Pishvaian MJ, Blais EM, Brody JR, et al. Overall survival in patients with pancreatic cancer receiving matched therapies following molecular profiling: a retrospective analysis of the know your tumor registry trial. Lancet Oncol. 2020;21:508–18. 10.1016/S1470-2045(20)30074-7. 32135080 10.1016/S1470-2045(20)30074-7 PMC7453743

[9] Mukai Y, Ueno H. Establishment and implementation of cancer genomic medicine in Japan. Cancer Sci. 2021;112:970–7. 10.1111/cas.14754. 33289217 10.1111/cas.14754 PMC7935799

[10] Kohno T, Kato M, Kohsaka S, et al. C-CAT: the national datacenter for cancer genomic medicine in Japan. Cancer Discov. 2022;12:2509–15. 10.1158/2159-8290.CD-22-0417. 36321305 10.1158/2159-8290.CD-22-0417 PMC9762342

[11] Sakakida T, Ishikawa T, Doi T, et al. Genomic landscape and clinical features of rare subtypes of pancreatic cancer: analysis with the national database of Japan. J Gastroenterol. 2023;58:575–85. 10.1007/s00535-023-01986-9. 37029223 10.1007/s00535-023-01986-9 PMC10199859

[12] Sakakida T, Ishikawa T, Doi T, et al. Genomic profile and clinical features of MSI-H and TMB-high pancreatic cancers: real-world data from C-CAT database. J Gastroenterol. 2023. 10.1007/s00535-023-02058-8. 38006445 10.1007/s00535-023-02058-8

[13] Sunami K, Naito Y, Aimono E, et al. The initial assessment of expert panel performance in core hospitals for cancer genomic medicine in Japan. Int J Clin Oncol. 2021;26:443–9. 10.1007/s10147-020-01844-1. 33385275 10.1007/s10147-020-01844-1 PMC7895780

[14] Florou V, Elliott A, Bailey MH, et al. Comparative genomic analysis of pancreatic acinar cell carcinoma (PACC) and pancreatic ductal adenocarcinoma (PDAC) unveils new actionable genomic aberrations in PACC. Clin Cancer Res. 2023;29:3408–17. 10.1158/1078-0432.Ccr-22-3724. 37266563 10.1158/1078-0432.CCR-22-3724

[15] Reissig TM, Tzianopoulos I, Liffers ST, et al. Smaller panel, similar results: genomic profiling and molecularly informed therapy in pancreatic cancer. ESMO Open. 2023;8: 101539. 10.1016/j.esmoop.2023.101539. 37148593 10.1016/j.esmoop.2023.101539 PMC10265614

[16] Fusco MJ, Saeed-Vafa D, Carballido EM, et al. Identification of targetable gene fusions and structural rearrangements to foster precision medicine in KRAS wild-type pancreatic cancer. JCO Precis Oncol. 2021. 10.1200/PO.20.00265. 34250383 10.1200/PO.20.00265 PMC8232071

[17] Philip PA, Azar I, Xiu J, et al. Molecular characterization of KRAS wild-type tumors in patients with pancreatic adenocarcinoma. Clin Cancer Res. 2022;28:2704–14. 10.1158/1078-0432.CCR-21-3581. 35302596 10.1158/1078-0432.CCR-21-3581 PMC9541577

[18] Naito Y, Aburatani H, Amano T, et al. Clinical practice guidance for next-generation sequencing in cancer diagnosis and treatment (edition 2.1). Int J Clin Oncol. 2021;26:233–83. 10.1007/s10147-020-01831-6. 33249514 10.1007/s10147-020-01831-6 PMC7819967

[19] Campbell BB, Light N, Fabrizio D, et al. Comprehensive analysis of hypermutation in human cancer. Cell. 2017;171(1042–56): e10. 10.1016/j.cell.2017.09.048. 10.1016/j.cell.2017.09.048 PMC5849393 29056344

[20] Strickler JH, Satake H, George TJ, et al. Sotorasib in KRAS p.G12C-mutated advanced pancreatic cancer. N Engl J Med. 2023;388:33–43. 10.1056/NEJMoa2208470. 36546651 10.1056/NEJMoa2208470 PMC10506456

[21] Bekaii-Saab TS, Yaeger R, Spira AI, et al. Adagrasib in advanced solid tumors harboring a KRAS(G12C) mutation. J Clin Oncol. 2023;41:4097–106. 10.1200/JCO.23.00434. 37099736 10.1200/JCO.23.00434 PMC10852394

[22] Hu C, Hart SN, Bamlet WR, et al. Prevalence of pathogenic mutations in cancer predisposition genes among pancreatic cancer patients. Cancer Epidemiol Biomark Prev. 2016;25:207–11. 10.1158/1055-9965.EPI-15-0455. 10.1158/1055-9965.EPI-15-0455 PMC4754121 26483394

[23] Takai E, Totoki Y, Nakamura H, et al. Clinical utility of circulating tumor DNA for molecular assessment in pancreatic cancer. Sci Rep. 2015;5:18425. 10.1038/srep18425. 26669280 10.1038/srep18425 PMC4680882

[24] Uson PLS Jr, Samadder NJ, Riegert-Johnson D, et al. Clinical impact of pathogenic germline variants in pancreatic cancer: results from a multicenter, prospective, universal genetic testing study. Clin Transl Gastroenterol. 2021;12: e00414. 10.14309/ctg.0000000000000414. 34620795 10.14309/ctg.0000000000000414 PMC8500569

[25] Peyraud F, Italiano A. Combined PARP inhibition and immune checkpoint therapy in solid tumors. Cancers (Basel). 2020. 10.3390/cancers12061502. 32526888 10.3390/cancers12061502 PMC7352466

[26] LaRose M, Manji GA, Bates SE. Beyond BRCA: diagnosis and management of homologous recombination repair deficient pancreatic cancer. Semin Oncol. 2024;51:36–44. 10.1053/j.seminoncol.2023.11.001. 38171988 10.1053/j.seminoncol.2023.11.001

[27] Hunter JC, Manandhar A, Carrasco MA, et al. Biochemical and structural analysis of common cancer-associated KRAS mutations. Mol Cancer Res. 2015;13:1325–35. 10.1158/1541-7786.MCR-15-0203. 26037647 10.1158/1541-7786.MCR-15-0203

[28] Witkiewicz AK, McMillan EA, Balaji U, et al. Whole-exome sequencing of pancreatic cancer defines genetic diversity and therapeutic targets. Nat Commun. 2015;6:6744. 10.1038/ncomms7744. 25855536 10.1038/ncomms7744 PMC4403382

[29] Yachida S, Jones S, Bozic I, et al. Distant metastasis occurs late during the genetic evolution of pancreatic cancer. Nature. 2010;467:1114–7. 10.1038/nature09515. 20981102 10.1038/nature09515 PMC3148940

[30] Mueller S, Engleitner T, Maresch R, et al. Evolutionary routes and KRAS dosage define pancreatic cancer phenotypes. Nature. 2018;554:62–8. 10.1038/nature25459. 29364867 10.1038/nature25459 PMC6097607

[31] Khaliq AM, Rajamohan M, Saeed O, et al. Spatial transcriptomic analysis of primary and metastatic pancreatic cancers highlights tumor microenvironmental heterogeneity. Nat Genet. 2024;56:2455–65. 10.1038/s41588-024-01914-4. 39294496 10.1038/s41588-024-01914-4

[32] Restle D, Dux J, Li X, et al. Organ-specific heterogeneity in tumor-infiltrating immune cells and cancer antigen expression in primary and autologous metastatic lung adenocarcinoma. J Immunother Cancer. 2023. 10.1136/jitc-2022-006609. 37349126 10.1136/jitc-2022-006609 PMC10314697

[33] Yang R, Qi Y, Kwan W, et al. Paired organoids from primary gastric cancer and lymphatic metastasis are useful for personalized medicine. J Transl Med. 2024;22:754. 10.1186/s12967-024-05512-0. 39135062 10.1186/s12967-024-05512-0 PMC11318189

[34] Ida H, Koyama T, Mizuno T, et al. Clinical utility of comprehensive genomic profiling tests for advanced or metastatic solid tumor in clinical practice. Cancer Sci. 2022;113:4300–10. 10.1111/cas.15586. 36106376 10.1111/cas.15586 PMC9746060

[35] Sokol ES, Pavlick D, Khiabanian H, et al. Pan-cancer analysis of BRCA1 and BRCA2 genomic alterations and their association with genomic instability as measured by genome-wide loss of heterozygosity. JCO Precis Oncol. 2020;4:442–65. 10.1200/po.19.00345. 32903788 10.1200/PO.19.00345 PMC7446440

